# Wireless Network for Assessing Temperature Load of Large-Scale Structures Under Fire Hazards

**DOI:** 10.3390/s19010065

**Published:** 2018-12-25

**Authors:** Robin E. Kim, Kiho In, Inhwan Yeo

**Affiliations:** Korea Institute of Civil Engineering and Building Technology; Goyang-Si 10223, Korea; kihoin@kict.re.kr (K.I.); yeo@kict.re.kr (I.Y.)

**Keywords:** fire safety engineering, performance-based fire protection design, wireless temperature sensor network

## Abstract

While the construction of high-rise buildings has become popular in big cities, an average of over 15,000 structure fires in those buildings are being reported in the United States. Especially because the fire in a building can result in a failure or even the collapse of the structure, assessing its integrity during and after the fire is of importance. Thus, in this paper, a framework with temperature sensors using wireless communication technology has been proposed. Associated hardware and software are carefully chosen and developed to provide an easy and effective solution for measuring fire load on large-scale structures during a fire. With an autonomous measurement system enabled, the key functions of the framework have been validated in a fire testing laboratory, using a real-scale steel column subject to standard fire. Unlike existing solutions of wireless temperature networks, the proposed solution can provide the user definable sampling frequencies based on the surface temperature and the means to assess the load redistribution of the structure due to fire loading in real-time. The results of the study show the great potential of using the developed framework for monitoring fire in a structure, allowing more accurate estimations of fire load in the design criteria, and advancing fire safety engineering.

## 1. Introduction

With the advancement of construction technology during the 20th century, high-rise buildings became more popular and taller [1]. According to a report from the Council on Tall Buildings and Urban Habitat (CTBUH), the total number of buildings over 200 m in the world exceeded 1000 in 2004, making a 392% increase from 2010 [2]. Such popularity trends have garnered significant attention in the safety of those high-rise buildings, including fire safety.

Losses from fires in high-rise buildings are high. In the years between 2007 and 2011, there were on average 15,000 high-rise building fires per year, resulting in death and injuries of over 540 civilians and direct property damage of $219 million (http://technokontrol.com/pdf/elpes/oshighrise.pdf). Ensuring lives and properties are safe from high-rise building fires is important, and thus, public focus has been directed in various fields including structure design, fire-resistance materials, evacuations, etc.

When the emphasis was to ensure structural integrity during fire hazards, researchers focused on the development and validation of the performance-based fire protection design (PBFPD). In PBFPD, buildings are designed to maintain their fundamental performance in a wide range of fire scenarios [3]. Compared to a conventional design approach, PBFPD is expected to meet the fire safety goals and performance requirements under fire scenarios and to be flexible and cost-effective in its design. However, the challenges of PBFPD are that it’s hard to define the code compliance and it lacks the necessary tools to quantify the design criteria [4].

Alvarez et al. summarized the shortcomings and challenges related to the applications of the current PBFPD of buildings [5]. Among those listed, one technical limitation of PBFPD addressed is that input (or the basic input assumptions) continues to require a conservative design approach [5,6]. In addition, Fitzgerald stated that a fire event in a building is far more complex, such that it is rather unpractical to treat its growth and propagation as an additional external load, as with an earthquake or wind, or to assume it follows a certain statistical distribution in time [7]. A technology that can assess the realistic fire load on a building is lacking in the field of fire engineering.

The popularity in using wireless communication, along with the advancement of sensing technology, has drawn wide use of those technologies in various engineering fields [8,9,10,11,12,13]. Compared to the traditional wired sensors and associated technologies, wireless communication is known to be easy to install and have less risk of data inundation, resulting in less cost for establishing a large-scale monitoring/sensing system [8,11,14,15]. Some attempts have been made to use wireless sensing technology in fire engineering. Fan et al. developed a wireless temperature monitoring system to evaluate the safety of a liquefied petroleum gas storage tank. In their work, ZigBee standards are used in the communication, and fiber optic high-temperature sensors are used to measure the tank temperature up to 125 °C, at sampling interval ranges from 1 sec to 24 hours [16]. Abaya et al. proposed an embedded wireless system to detect fire and flame in real time [17]. The authors’ major focus of the listed research is to detect fire at an early stage to help residents to evacuate or to reduce the impact of the fire.

When it comes to a fire in a structure, the safety of the building during the fire is of concern [18]. Hu et al. stated that due to load redistribution on the main components of the building, the structure can fail or even collapse. Thus, some efforts have also been made to monitor the surface temperature of a structure using wireless sensors [18]. Cheng et al. developed a wireless passive temperature sensor based on a reflective patch and demonstrated that the device can measure surface temperature up to 1050 °C [19]. Regarding the application of wireless temperature sensors in the monitoring of a structure, Kim et al. developed a passive wireless temperature sensor. Developed sensors are able to measure a change of temperature with a range of −10 °C to 100 °C [20]. As listed, several researchers have developed wireless sensors that can measure the thermal loads of a structure. However, an integrated system that can measure the fire load in a global manner and allow understanding of structural response subject to the fire loading is still lacking.

Thus, in this paper, a framework that can assess the global behavior of a building structure during a fire event using wireless communication is proposed. In the proposed framework, an integrated temperature system that can automatically detect and measure surface temperature up to 1000 °C is developed. To validate the developed framework, a real-scale column was developed and tested in a column furnace using standard fire, which is the time‒temperature curve used in fire-resistance tests [3]. The measured temperature can further be used for assessing the global behavior of the structure from a stand-alone health monitoring system. The primary focus of the presented work being to propose an integrated framework for monitoring structures under fire using wireless communication, real-scale steel columns were developed and tested to validate the system. The results of the proposed system show great potential in facilitating wireless sensing technology for monitoring a structure during a fire event. Furthermore, the proposed approach can provide a tool for quantifying the design criteria of PBFPD, ensuring the safety of high-rise buildings.

## 2. Requirements for a Temperature Sensor Network for a Steel Structure

This section discusses the requirements of a wireless temperature sensor network for assessing temperature loading of a structure. The primary focus of the application is for a typical steel structure, although the framework can accommodate any type of material or structure. In addition, the proposed system aims to be flexible such that it can be applied to high-rise or large building structures.

When a steel structure is exposed to a fire, the surface temperature rises and, at the same time, loses strength and stiffness, resulting in a possible deformation or failure of the structure depending on the initial loading or condition [3]. For example, when the surface temperature exceeds 400 °C, about 30% of elasticity is lost [21]. The limiting temperature of a steel structure, at which the element is expected to fail, is known to be around 600 °C [22]. However, some full-scale tests and real-fire behavior reveal that the behavior of a steel structure under fire highly depends on the system level factor rather than element level factors [3,23]. Thus, to capture the global behavior of a steel structure, the system needs to be capable of measuring temperatures up to around 700 °C and at the structure level.

Another consideration is that when the structure becomes larger, the surface domain that needs to monitor fire events also increases. Thus, a strategy that can maximize the benefit of using wireless sensing technology, in that it can lessen the risk of data inundation, needs to be devised. For example, a sensing part of the framework has to be capable of handling raw data before transmitting it to the storage part. In addition, an autonomous system to the wireless network should be implemented to detect any fire hazards at a certain interval.

Finally, a framework needs to be able to survive in harsh environments. Although the radio transmission itself is not affected by high temperatures, the physical devices, such as the radio transmitter, must withstand the harsh environment. As presented in Boan (2007), radio propagation during a fire can be greatly degraded [24]. In summary, for a successful framework, the integrated system needs to satisfy the following requirements:

- Capable of measuring surface temperature up to 700 °C
- Enabled with an autonomous measurement system
- Adjustable measurement intervals with respect to its circumstances
- A standalone solution for radio transmitting under high temperature

## 3. Wireless Temperature Network Framework for Steel Buildings

This section describes a proposed framework for temperature monitoring of a steel structure and associated hardware and software developments, which aimed to satisfy the requirements listed in the earlier section. Strategies for the overall framework are first discussed. Then, associated development in the network components, including sensor and data transmission hardware, follow. Finally, the software development and real-scale instrumentation are presented.

### 3.1. Wireless Network Framework

**Figure 1:**
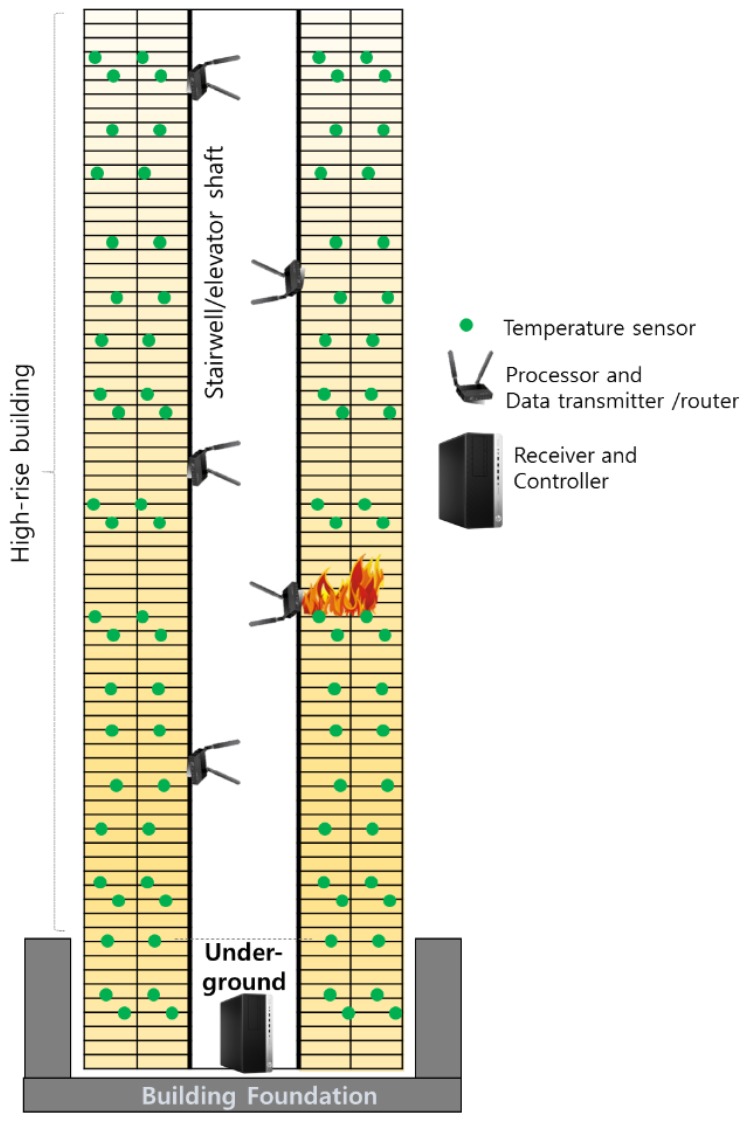
The architecture of the proposed framework in a high-rise building.

In the proposed wireless temperature network, the framework is composed of three parts as shown in Figure 1: (1) temperature sensors (e.g., thermocouple); (2) processors and data transmitters; and (3) a receiver and controller. In the scheme, sensors are located at the main members of the structure, such as columns and girders. The processors/data transmitters, to which sensors are wire connected, are assumed to be located at a stairwell or an elevator shaft. Although a stairwell can still suffer from signal degradation due to stair steps, such vertical open spaces have the merits of line-of-sight links for vertically delivering data. The controller can be considered as the main PC, which can be located in the machine room of the building. The processors/data transmitters can process the collected data and decide whether to transmit it to the controller. The overall objective of the design scheme is to be flexible, expandable, and reliable in terms of wireless communication.

**Figure 2:**
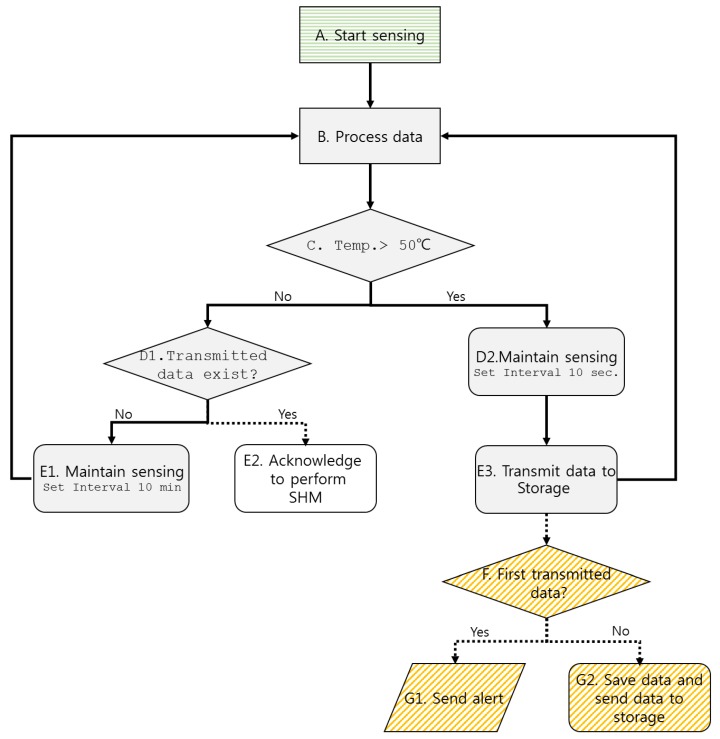
The flow chart of the proposed framework during/after a fire on a building.

More detailed processes on how the framework operates are illustrated in Figure 2. When a temperature sensor is installed, the sensor starts measuring the surface temperature (A. Start sensing in Figure 2). At the same time, the processor checks the first read-in data (B. Process) to check if the surface temperature exceeds 50 °C, which is higher than ambient surface temperature, which is usually around 20 °C (C. Temp. >50 °C). Although the temperature threshold is set at 50 °C, the value is highly user definable. If the first measured temperature is less than 50 °C (D1. Transmitted data exist), the transmitter acknowledges the receiver to set the measurement interval to 10 min (E1. Maintain sensing). It is important to note that with the 10 min setting, the worst case is that steel surface temperature with standard fire following ISO 834 [25] will reach around 150 °C, implying it the fire is still in the early stages. However, if, on the other hand, the first measured temperature is over 50 °C, the measuring interval is set to 10 s, and the controller sends an alert message on its dashboards (A-B-C-D2-E3-F-G1 process in Figure 2). When standard fire, such as ISO 834, occurs in a steel structure, it takes more than 50 min until the average surface temperature reaches 800 °C. Such a fact validates setting the measurement interval to 10 s, although shorter intervals can also be selected at the user’s end. Sequential data at the processor/data transmitter side continue to be transmitted to the receiver to be saved (A-B-C-D2-E3-F-G2). When the surface temperature cools down to less than 50 °C, the system recognizes it as the termination of the fire event. Then, the system starts to utilize the collected data to understand the global behavior of the structure under the recent fire loading (A-B-C-D1-E2). The scope of the framework presented in the paper is limited to during the fire loading event, excluding a description of the global understanding of the structure, which is to be performed in the post-fire event. Thus, the focus is only on proposing the schematic framework for collecting surface temperature loading of a structure using wireless communication and its applications.

### 3.2. Network Hardware Components

This section describes the hardware components used for performing the framework illustrated in Figure 2. The hardware is composed of three main components: (1) temperature sensor, (2) data converter, and (3) wireless module. Regarding the temperature sensor, K-type thermocouples were selected from among various types of temperature sensors. While some other common types of thermocouples are compared in Table 1, the selected type is one of the most common thermocouples with the widest operating temperature range (from −200 °C to 1000 °C). When the outer part is insulated with a metal sheath, the thermocouple (http://www.thermometricscorp.com/thertypk.html (accessed in June 2018)) is known to resist temperatures up to 1260 °C. A 0.65 mm diameter K-type thermocouple is used throughout this paper, although any type can be selected. The selected thermocouple is therefore robust and reliable enough to measure the surface temperature of a steel structure.

Then, a commercialized thermocouple data converter, PT8TCS (Profibus I/O Modules) (PROCON electronics, Sydney, Austrailia, www.proconel.com (accessed in June 2018)), was selected. PT8TCS has 8 isolated input channels, can adjust its sampling from 0.63 Hz, and shows the resolution of 0.1 °C. The converter has a default IP address that can easily be hooked up to a local network or the wireless transmitting module. Thus, in this paper, a wireless router (ACKSYS, AirLink www.acksys.fr/produits (accessed in August 2018)) is connected to the data converter to transfer collected data to the receiver in a robust manner. The selected hardware components are aimed to be widely accepted for industrial Wi-Fi access and compliant with IEEE 802.11 a/b/g/n Wi-Fi standards (2.4 and 5 GHz), offering up to 400 Mbps radio data rate. The system requires a 10 V power supply for each node. The system is assumed to be located inside a building where a stable power supply is available in a normal scenario. However, a UPC with fire-proof coverage will be needed to provide adequate power to the wireless system during a fire in reality. Thus, the selected set of hardware allows flexible and robust data collection and transmission so that the developed framework can be reliably employed on a large-scale structure.

### 3.3. Software Development and Instrumentation Setup

**Figure 3:**
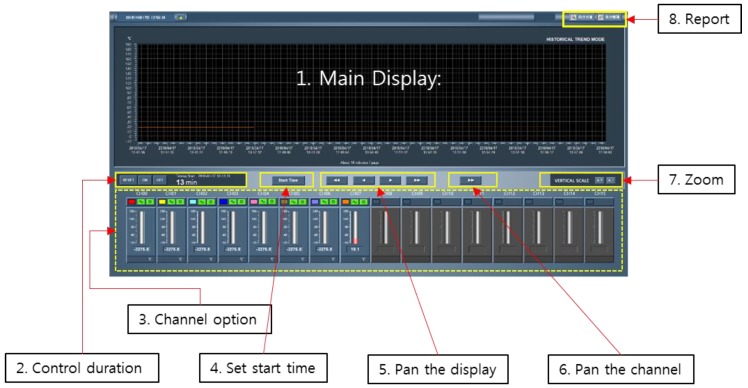
User interface showing measured temperature history from the networks.

To operate the framework described in Figure 2, associated software was developed. The main dashboard at the users’ end is illustrated in Figure 3. In this specific example, only a single PT8TCS is connected in the network, i.e., eight sensing channels. The main functions of the software can be summarized as below:

- It is capable of displaying the temperature history of the individual channel.
- The number channels can be expanded as more data converters participate in the network.
- The sampling rate can be chosen from 1 sec to 10 min, although the default interval is 10 min in the beginning.
- The operating computer time is shared in the network so that it can set the global time to save measured data.
- It is enabled with an alarm light when a certain threshold is exceeded in at least one channel.

Detailed functions are as shown in Figure 3, which includes the following: (1) the main display to show the temperature‒time history of the selected channels. (2) The display controller can reset the display and turn the display off and on when needed. The duration of the display is also shown on a minute scale. (3) The channel option panel shows the current temperature at each channel in terms of Celsius. Furthermore, using the panel, the user can change the color of a channel and decide to show or not show the channel on the display. (4) Using the set start time button, the user can choose the absolute time to start the measurement in the system. (5) The pan the display option can pan the main display to show whatever the user decides to show. (6) The pan the channel button can pan the channel option when the channel is more than 16 channels and needs to be controlled. (7) The zoom button can zoom in and out on the temperature‒time history on the main display. (8) Finally, using the report button, the user can export a report.

Although the developed software is capable of hooking up several data converters, the software is lacking in its ability to synchronize collected data from various converters. Because the sequential data transmitting delay time is ignored due to a small sized network, to be more expandable for larger-scale structures, the data synchronization function still needs to be improved in the software.

**Figure 4:**
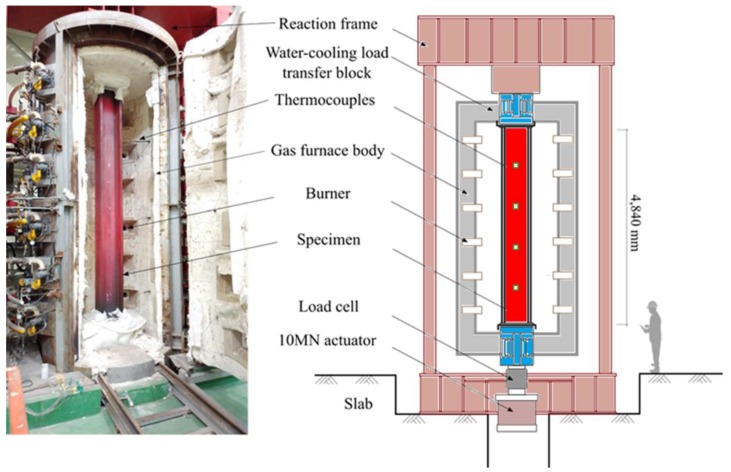
Benchmark experimental setup with column furnace and steel column [26].

To validate the main functions of the framework, a benchmark problem from Wang et al. (2018) was selected. In their work, a reference structure is presented, which is a real-scale four-story steel frame building. To evaluate the global response of the structure, a middle column on the first floor was manufactured and tested in a column furnace, as shown in Figure 4. The furnace is capable of applying an external compressive load up to 1000 tons and any type of fire scenario. For the testing specimen, the length was about 4.84 m with a section size of W14x311 [26]. The manufactured column underwent the standard fire curve from ISO 834 [25], along with additional external loading (up to around 5 MN) from the structural analysis [26]. While the scope of this paper is limited to presenting the operation of the developed framework, the benchmark result will be used for assessing the PBFPD of the four-story building under fire and external loadings.

**Figure 5:**
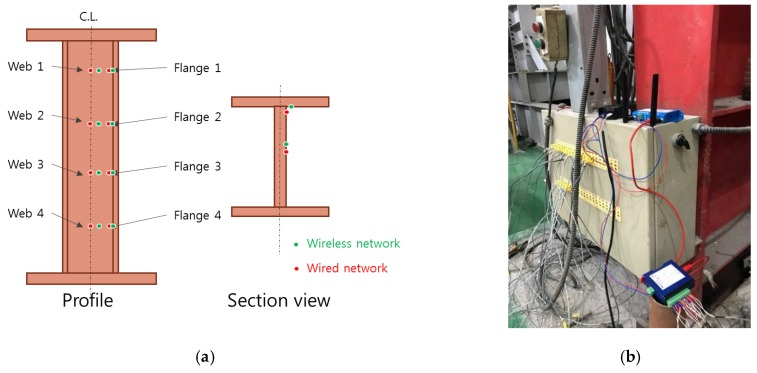
Sensor topology and network installation for the network validation: (**a**) sensor locations, (**b**) converter and transmitter.

The installed thermocouples are illustrated in Figure 5a. To be comparable with a wired sensor network, a total of 16 sensors for both wired and wireless communication systems were installed. Along its length, four locations were selected to have about 1 m distance from the bottom. Notations of the sensors (for both wireless and wired) started from the very top such that the top two locations were noted with Web1 (center) and Flange1 (outer side) and so on. To reduce the interference of the two nearby thermocouples, the distance between the wireless network sensors and the reference sensors (wired network) was about 1 cm. During a fire, the sampling rate for the wired sensors was set to be 1 min, whereas the wireless network could sample at 10 s when the temperature was high. Furthermore, throughout the fire test, the furnace door was closed tightly and sealed, and the converter and transmitter were placed outside the furnace (Figure 5b).

**Figure 6:**
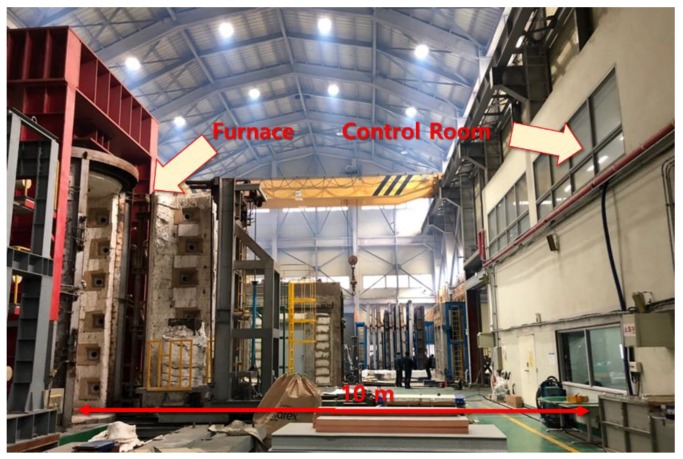
Experimental radio environment in the laboratory; a transmitter antenna is 1 m above the ground and a receiver is located in the control room.

More detailed descriptions of the radio environment for the experiment are shown in Figure 6. The column furnace used in this study is located in a large indoor laboratory. In this space, especially during the test, no other wireless radio was turned on, including Wi-Fi. The selected properties of the router were 2.4 GHz TX Power, 300 Mbps data rate, and 3 dBi antennas. The transmitter was located 1 m above the ground near the furnace. The transmitting router was at room temperature, with no nearby specific heat generated. The receiving part was located in a control room, as shown in Figure 6. The control room was about a half-story above the furnace, which was blocked with 4 cm thick glass wall. Nominally, the distance from the furnace was about 10 m.

## 4. Experimental Results 

The standard fire test was performed until the average surface temperature exceeded 700 °C, which took about 45 min. For the first 28 min, the radio receiver/controller was located in the control room. Then, to check the radio transmissivity, the radio receiver/controller was moved downstairs, where the floor is made of a 12 cm thick concrete slab. Then, the receiver was moved back to the original room at the end of the test.

**Figure 7:**
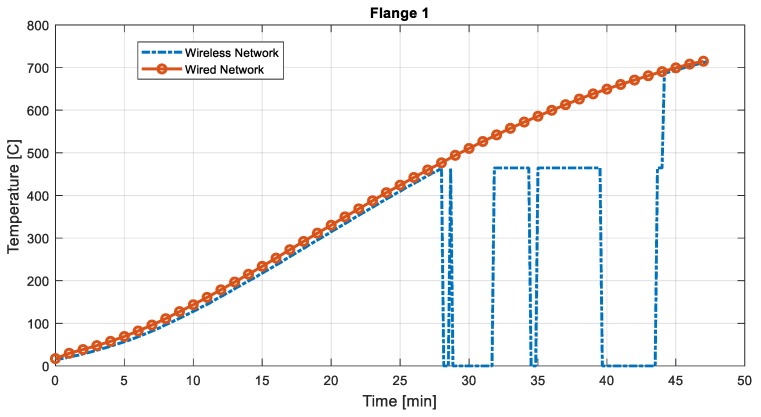
Temperature and time history measured at flange1–full time.

The temperature reading during the entire test period is illustrated in Figure 7. While the receiver was located inside the room, the temperature log shows a similar reading to that of the wired network. However, when the receiver was blocked with the concrete slab, the data could not be received, therefore only showing the most recent meaningful temperature data. As soon as the radio signal became available, temperature readings recovered to match the wired network. Such a result reveals the importance of selecting locations for the radio transmitter and receivers when wireless communication is used.

**Figure 8:**
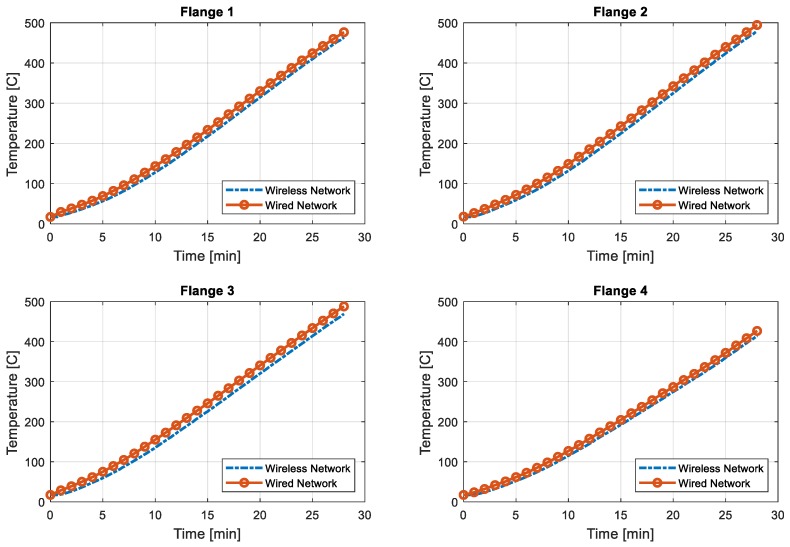
Temperature comparison for flange—first 28 min.

**Figure 9:**
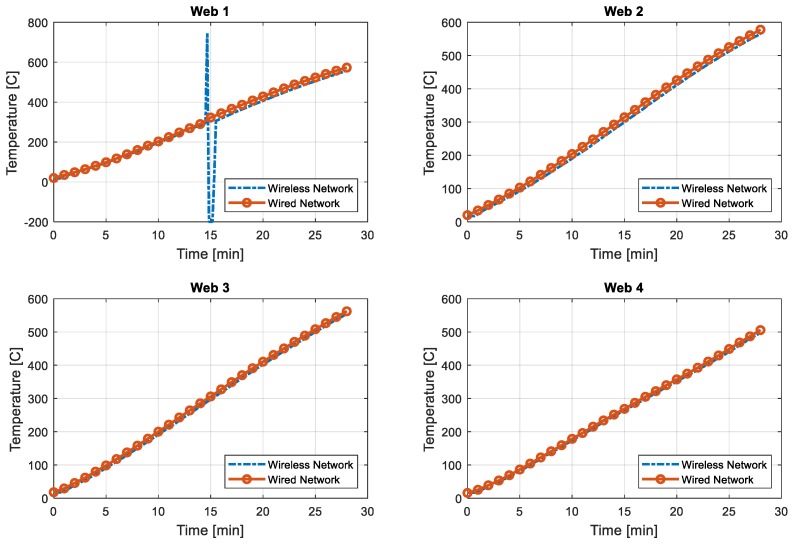
Temperature comparison for web—first 28 min.

To illustrate the accuracy of the wireless network effectively, only the first 28 min of data are compared in Figure 8 and Figure 9. Figure 8 displays the data from the flange, while Figure 9 shows the web temperature. At the flange side, the temperature from the wireless network appears to always show a smaller reading than that of the wired network. When the web temperature is compared, the wireless network shows a similar value to that in the wired network. Slight differences in the temperature log may originate from locational difference, not from network or device error. It should be noted that, as shown in Figure 6, the thermocouples for the wired network were installed on the web, while the sensors for the wireless network were installed on the flange. Although the flange and the web were made of the same material, locational difference or heat conduction rate differences may have resulted in such errors.

In Figure 9, Web1 showed an unexpected peak at around 15 min. The temperature reading increased to 744 °C and then to −200 °C. Seeing the phenomenon only once in the channel, it recovered to the accurate reading, and no other channels showed such a behavior. The error may have come from wireless data conflict, corruption, or delay. However, knowing that there was no other wireless radio exited during the test, one possibility may have come during the radio packet conversion at the receiving or transmitting part. To further reliably use the wireless communication, a larger test needs to be performed to identify exactly which factor could cause failure in data transmission. In addition, in this version, no error correction is used. Thus, the software needs to be improved to detect and correct erroneous signals and to resend the request message to obtain the intact data.

**Figure 10:**
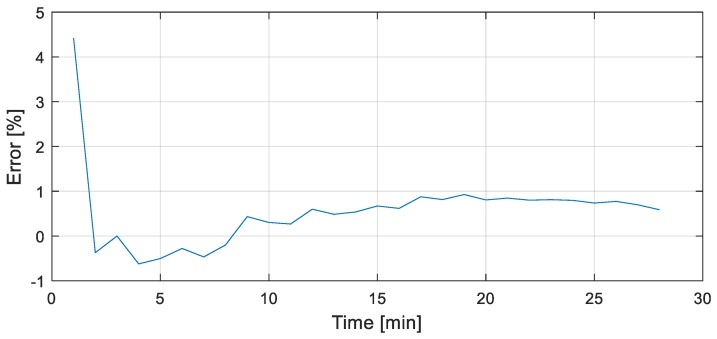
Temperature comparison—[location ID: web2].

Finally, to quantitatively compare the wireless and wired network, Figure 10 shows the error in the wireless network by percentage. The chosen location is Web2. Because the wired and wireless networks were not data synchronized, meaning that data sampled timing for the two systems were different, data from the wireless network was resampled manually. As can be seen from Figure 10, the error is within ±1% throughout most of the testing period. The first data show larger differences may have come from the combination of factors such as: (1) initial offset, (2) initial nonlinearly in the furnace operation, and (3) data synchronization error, etc. When temperature increases, the error seems to be standardized, showing good accuracy between those two systems.

## 5. Conclusions

In this paper, a framework for a temperature sensor using wireless communication was developed. The developed set of hardware, software, and algorithm has merits in the field of fire engineering, providing an easy tool for measuring fire load on a structure. The developed tool can further allow a quantitative evaluation of a structure under fire events, which has not yet been fully provided. To meet the requirements of measuring fire loads on steel structures, a set of networks including sensors, data converter, and wireless communication device, were selected carefully. Then, software was developed to effectively control the network. To validate the applicability of the proposed framework, a real-scale steel column was instrumented with sixteen thermocouples that were connected to the wired and wireless system. Then the column was placed in a furnace for over 45 min of standard fire testing, ISO 834 [25]. The results of the test showed good accuracy of the developed framework. A summary of the major findings of the presented work is as follows:

- As compared to traditional wired sensors, the wireless network has the potential of being cost-effective, especially when the scale of a structure becomes larger.
- An autonomous system that selects the data sampling rate based on its temperature is developed in preference to a traditional approach by making the system smart and also allowing the handling of a large number of sensors easily.
- However, when dealing with wireless communication, a careful selection of the radio propagation paths is needed to avoid data losses from data corruption, delay, blockage, etc.
- Especially, further testing of the system on a real structure and inclusion of error compensating strategies are needed to ensure a reliable radio communication.
- To further use the developed framework reliably, more improvements in the software are needed such as detecting erroneous data, handling transmission error, delay, etc.

The results of the proposed system show great potential in facilitating wireless sensing technology in monitoring structural fires. Using the developed framework, future applications can be made including the following: (1) measuring the fire load on in-service structures and aiding the advancement and evaluation of the performance-based design, (2) monitoring the structural temperature and its global behavior when a structure is subject to a local fire, and (3) developing real-time evacuation maps for residents during fire events.

## Figures and Tables

**Table 1:** Measurable temperature range of various common types of thermocouples thermocouple (http://www.thermometricscorp.com/thertypk.html (accessed in June 2018)).

Type	Temperature Range (°C)
K-type	−200~1250
J-type	0~750
E-type	−200~900
T-type	−250~350
